# Effect of strain relaxation on performance of InGaN/GaN green LEDs grown on 4-inch sapphire substrate with sputtered AlN nucleation layer

**DOI:** 10.1038/s41598-019-40120-9

**Published:** 2019-03-05

**Authors:** Hongpo Hu, Shengjun Zhou, Hui Wan, Xingtong Liu, Ning Li, Haohao Xu

**Affiliations:** 1 Center for Photonics and Semiconductors, School of Power and Mechanical Engineering, Wuhan University, Wuhan, 430072 China; 2 State Key Laboratory of Applied Optics, Changchun Institute of Optics, Fine Mechanics and Physics, Chinese Academy of Sciences, Changchun, 130033 China

## Abstract

Here we demonstrate high-brightness InGaN/GaN green light emitting diodes (LEDs) with *in-situ* low-temperature GaN (LT-GaN) nucleation layer (NL) and *ex-situ* sputtered AlN NL on 4-inch patterned sapphire substrate. Compared to green LEDs on LT-GaN (19 nm)/sapphire template, green LEDs on sputtered AlN (19 nm)/template has better crystal quality while larger in-plane compressive strain. As a result, the external quantum efficiency (EQE) of green LEDs on sputtered AlN (19 nm)/sapphire template is lower than that of green LEDs on LT-GaN (19 nm)/sapphire template due to strain-induced quantum-confined Stark effect (QCSE). We show that the in-plane compressive strain of green LEDs on sputtered AlN/sapphire templates can be manipulated by changing thickness of the sputtered AlN NL. As the thickness of sputtered AlN NL changes from 19 nm to 40 nm, the green LED on sputtered AlN (33 nm)/sapphire template exhibits the lowest in-plane compressive stress and the highest EQE. At 20 A/cm^2^, the EQE of 526 nm green LEDs on sputtered AlN (33 nm)/sapphire template is 36.4%, about 6.1% larger than that of the green LED on LT-GaN (19 nm)/sapphire template. Our experimental data suggest that high-efficiency green LEDs can be realized by growing InGaN/GaN multiple quantum wells (MQWs) on sputtered AlN/sapphire template with reduced in-plane compressive strain and improved crystal quality.

## Introduction

The combination of high-efficiency red, green, and blue (RGB) light emitting diodes (LEDs) can produce more efficient white LEDs by eliminating the Stoke’s losses related to the phosphor^1,2^. A peak external quantum efficiency (EQE) exceeding 83% has been reported for the GaN-based blue LEDs^3^. However, the EQE of the InGaN/GaN green LEDs is still relatively poor. The low efficiency of green LEDs, a phenomenon known as the ‘green gap’, is a critical factor hindering the realization of high-performance RGB white LEDs in solid-state lighting^4–7^. In general, there are two main reasons for the ‘green gap’ phenomenon. On one hand, InGaN/GaN MQWs with higher indium (In) composition are required for LEDs emitting at green spectra. Due to the 11% lattice mismatch between InN and GaN, the c-plane InGaN/GaN multiple quantum wells (MQWs) grown on sapphire substrate are subject to in-plane compressive stress and suffer from quantum confined Stark effect (QCSE) induced by the strain-induced piezoelectric field^8–16^. The QCSE limits the internal quantum efficiency (IQE) of green LEDs by reducing the overlap integral between the electron and hole wave functions, leading to a lower radiative recombination efficiency.On the other hand, low growth temperatures is necessary to incorporate higher In-concentration in the InGaN/GaN MQWs for achieving green emission, which introduces impurities and defects, resulting in an increase in Shockley-Read-Hall (SRH) non-radiative recombination^17^.

Several solutions have been proposed to enhance the efficiency of green LEDs including nanopatterned sapphire substrate^18^, staggered InGaN QW^19,20^, non- and semi-polar QWs^21–23^, lattice-matched AlGaInN barrier^24^, and AlGaN or AlInN interlayers^25–28^. An *in-situ* low-temperature GaN (LT-GaN) or AlN nucleation layer (NL) between the high-temperature GaN epilayers and substrate is generally introduced to reduce defect density in GaN film^29,30^. The *ex-situ* sputtered AlN NL is another choice for green LEDs with advantages over LT-GaN/AlN NL^31,32^. First, the sputtered AlN NL enables one-step growth of GaN film to reduce growth time and thermal cycles of epitaxy. Second, a low threading dislocation density (TDD) of GaN film can be obtained using sputtered AlN/sapphire template. However, Miyoshi *et al*. demonstrated that the InGaN/GaN MQWs grown on AlN/sapphire template has a larger in-plane compressive strain than those grown on LT-GaN/sapphire template^33^. The larger in-plane compressive strain in the InGaN QWs would induce a larger piezoelectric polarization electric field, resulting in a more severe QCSE for LED on sputtered AlN/sapphire template. Therefore, to realize high-efficiency green LEDs on sputtered AlN/sapphire template, the in-plane compressive strain in the green InGaN/GaN MQWs should be manipulated to alleviate the detrimental effect of the QCSE.

In this paper, we demonstrate that the in-plane compressive strain of green LEDs on sputtered AlN/sapphire templates can be modified by changing thickness of the sputtered AlN NL. Compared to the green LED on LT-GaN (19 nm)/sapphire template, the green LED on sputtered AlN (19 nm)/template has larger in-plane compressive strain and thus lower EQE. However, at an optimal thickness of sputtered AlN NL (33 nm), the in-plane compressive strain of green LED can be minimized. These findings reveal that in-plane compressive strain of green LEDs on LT-GaN and sputtered AlN/sapphire templates plays a dominant role in determining device efficiency, while the thickness manipulation of *ex-situ* sputtered AlN NL can be an effective method to relax in-plane compressive strain. As a result, the EQE of 526 nm green LED on sputtered AlN (33 nm)/sapphire template is 6.1% larger than that of the green LED on LT-GaN (19 nm)/sapphire template at 20 A/cm^2^. The higher efficiency of green LED on sputtered AlN (33 nm)/sapphire template accompanied with reduced growth time and thermal cycles of epitaxy suggest the *ex-situ* sputtered AlN NL may be a superior alternative solution relative to the *in-situ* LT-GaN NL.

## Results and Discussion

**Figure 1:**
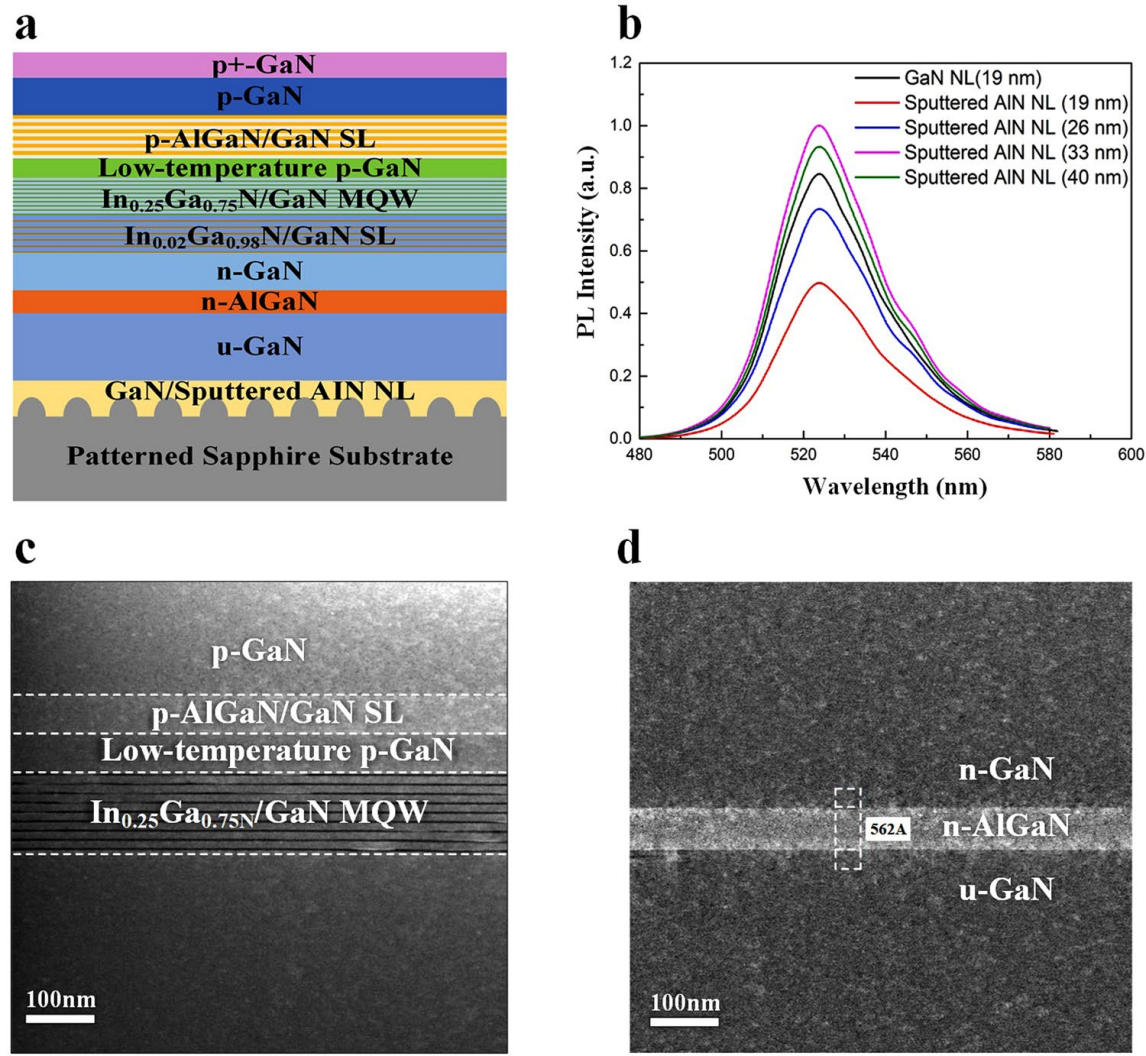
(**a**) Schematic representation of InGaN/GaN the green LED epitaxial structure. (**b**) PL spectra (T = 300 K) of the green LED on LT-GaN and sputtered AlN/sapphire templates. (**c** and **d**) Cross-sectional TEM images of the green LED epitaxial structure.

Figure 1(a) shows schematic representation of the green LEDs epitaxial structure. Photoluminescence (PL) spectra of the green LED measured using a 405 nm laser diode are described in Fig. 1(b). As the sputtered AlN NL thickness increases, the PL peak intensity of green LEDs on sputtered AlN/sapphire templates increases and reaches a maximum value at a thickness of 33 nm, thereafter decreasing with a further increase of the thickness to 40 nm. While the PL peak intensity of green LEDs on sputtered AlN (19 nm)/sapphire template is weaker than that of green LEDs on LT-GaN (19 nm)/sapphire template, the PL peak intensity of green LEDs on sputtered AlN (33 nm)/sapphire template is approximately 1.02 times stronger than that of the green LED on LT-GaN (19 nm)/sapphire template. Such great improvement shows the advantage of using optimal AlN NL thickness for high-efficiency green LEDs. Figure 1(c,d) show cross-sectional TEM images of green LEDs. Thickness fluctuations are observed through a single QW, which could explain the broad PL spectra for these green LEDs.

**Figure 2:**
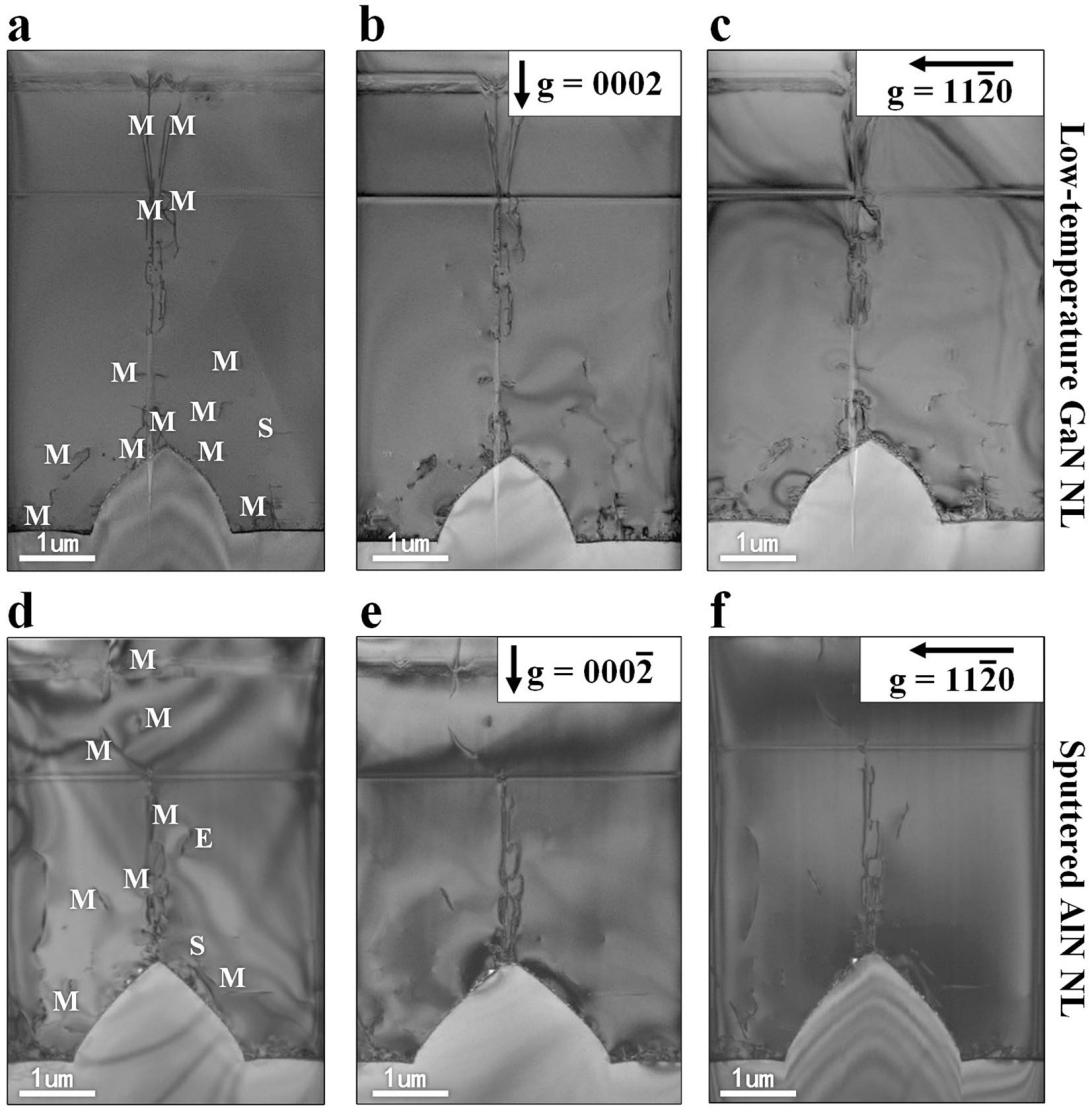
Cross-sectional TEM images of green LEDs cut in the (10–10) zone axis. (**a**,**d**) Cross-sectional BF TEM images showing screw (S), edge (E), and mixed (M) dislocations. (**b**,**e**) Cross-sectional BF TEM images with g = (0002) showing screw and mixed type dislocations. (**c**,**f**) Cross-sectional BF TEM images with g = (11–20) showing edge and mixed type dislocations.

We compared TDD of green LEDs on LT-GaN (19 nm) and sputtered AlN (19 nm)/sapphire templates. Screw (a-type), edge (c-type), and mixed-type (a+c-type) dislocations have a Burgers vector b = <0001>, b = 1/3 <11–20>, and b = 1/3 <11–23>, respectively. Cross section was cut out of the green LED to identify the screw, edge, and mixed type dislocations in the (10–10) zone axis using two perpendicular diffraction vectors g = (0002) and g = (11–20). Figure 2(a,d) demonstrate the cross-sectional bright-field (BF) TEM images of green LEDs on LT-GaN (19 nm) and sputtered AlN (19 nm)/sapphire templates, where the screw, edge, and mixed type dislocations are marked in S, E, and M, respectively. The cross-sectional BF TEM images taken under the condition of g = (0002) (Fig. 2b,e) reveal the screw and mixed dislocations and those taken under the condition of g = (11–20) (Fig. 2c and f) reveal the edge and mixed dislocations. The screw dislocation should be in contrast for g = (0002) and disappear for the perpendicular diffraction vector g = (11–20), while the edge dislocations should be in contrast for g = (11–20) and disappear for the perpendicular diffraction vector g = (0002). By comparing same areas of the sample with two perpendicular diffraction vectors, most of TDs can be identified as mixed dislocation since they should be in contrast for both these vectors. We can clearly see that the green LED on sputtered AlN/sapphire template has a lower TDD than the green LED on LT-GaN/sapphire template.

**Figure 3:**
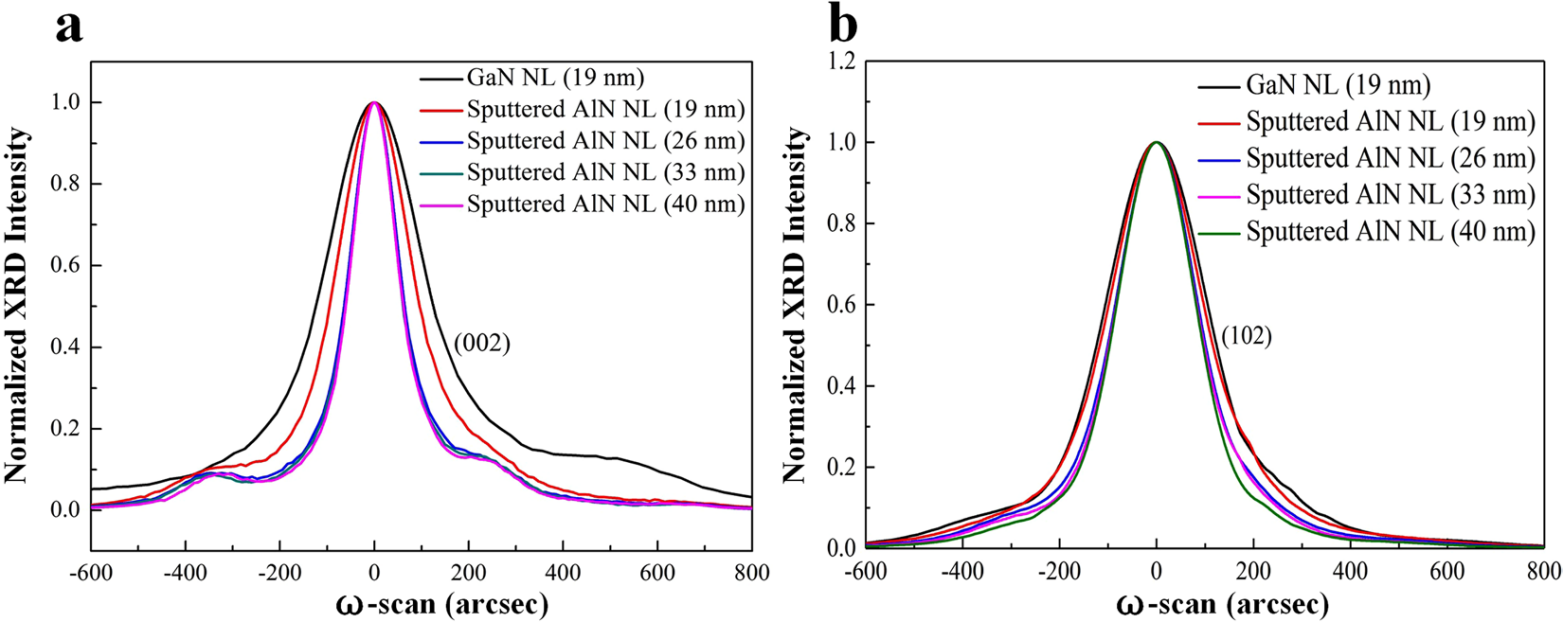
(**a**) Symmetric (002) and (**b**) asymmetric (102) reflections for green LEDs on LT-GaN and sputtered AlN/sapphire templates.

To investigate the effects of the NLs on crystal quality of green LEDs, we performed high-resolution X-ray diffraction (XRD) rocking curves measurement. Figure 3 compares the full widths at half-maximum (FWHMs) of symmetric (002) and asymmetric (102) reflections for green LEDs on LT-GaN and sputtered AlN/sapphire templates. The (002) plane X-ray rocking curve FWHMs are 249.1 and 190.6 arcsec for the green LED on LT-GaN (19 nm)/sapphire template and the green LED on sputtered AlN (19 nm)/sapphire template, respectively. The (102) plane X-ray rocking curve FWHMs are 247.6 and 233.4 arcsec for the green LED on LT-GaN (19 nm)/sapphire template and the green LED on sputtered AlN (19 nm)/sapphire template, respectively. Compared to the green LED on LT-GaN (19 nm)/sapphire template, we observed a decrease of the (002) FWHM and (102) FWHM for the green LED on sputtered AlN (19 nm)/sapphire template. It is well known that the FWHMs of symmetric (002) and asymmetric (102) reflections are affected by screw and edge dislocation densities, respectively^34,35^. According to the measured (002) and (102) FWHMs, the screw dislocation densities of green LEDs on LT-GaN (19 nm) and sputtered AlN (19 nm)/sapphire templates are determined to be 1.25 × 10^8^ and 7.29 × 10^7^ cm^−2^, respectively; the edge dislocation densities of green LEDs on LT-GaN (19 nm) and sputtered AlN (19 nm)/sapphire templates are determined to be 3.25 × 10^8^ and 2.89 × 10^8^ cm^−2^, respectively. These results indicated that the screw dislocation density of the green LED on sputtered AlN (19 nm)/sapphire template was much lower than that of the green LED on sputtered AlN (19 nm)/sapphire template.

We also analyzed the effect of sputtered AlN NL thickness on crystal quality of green LEDs. When the thickness of sputtered AlN NL was varied from 19 nm to 40 nm (19, 26, 33 and 40 nm), the (002) FWHMs of green LEDs on sputtered AlN/sapphire templates were 190.6, 128.3, 121.9, and 122.1 arcsec; the (102) FWHMs of green LEDs on sputtered AlN/sapphire templates were 233.4, 202.3, 197.4, and 192.6 arcsec. The corresponding screw dislocation densities of green LEDs on sputtered AlN/sapphire templates were 7.29 × 10^7^, 3.31 × 10^7^, 2.98 × 10^7^, and 2.99 × 10^7^ cm^−2^; the corresponding edge dislocation densities of green LEDs on sputtered AlN/sapphire templates were 2.89 × 10^8^, 2.17 × 10^8^, 2.07 × 10^8^, and 1.97 × 10^8^ cm^−2^. This revealed that the TDD of green LEDs on sputtered AlN/sapphire template would be significantly increased when using a thin sputtered AlN NL (19 nm).

**Figure 4:**
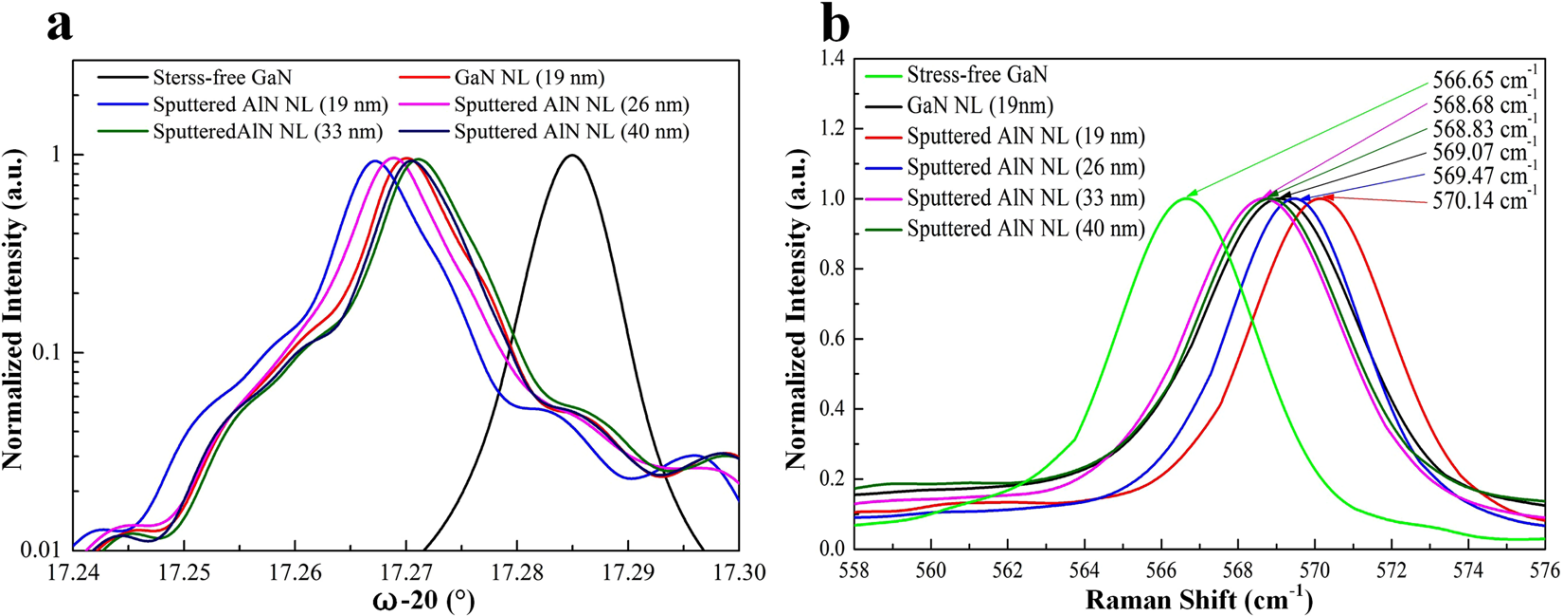
(**a**) XRD *ω*-2*θ* scans and (**b**) Raman spectra for green LEDs on LT-GaN and sputtered AlN/sapphire templates.

High-resolution XRD was exploited to extract out-of-plane lattice parameter c and strain state of green LEDs on LT-GaN and sputtered AlN/sapphire templates. Figure 4(a) shows *ω*-2*θ* scans through (0002) reflection of GaN for green LEDs. The lattice constants c for the green LEDs with LT-GaN NL (19 nm) and with various thicknesses of sputtered AlN NLs (19, 26, 33, and 40 nm) on sapphire substrates are estimated to be 5.189500 Å, 5.190320 Å, 5.189862 Å, 5.189186 Å, and 5.189316 Å, respectively. The lattice contact c for the stress-free GaN bulk substrate is estimated to be 5.184980 Å. We find that the lattice constant c for the green LED on LT-GaN (19 nm)/sapphire template is smaller than the value for the green LED on sputtered AlN (19 nm)/sapphire template. Furthermore, the lattice constants *c* for the green LEDs on sputtered AlN/sapphire template are found to vary with the thickness of the sputtered AlN NL. According to the Poisson effect, the expansion of out-of-plane lattice indicates the shrinkage of in-plane lattices at the same time. Hence, the larger lattice constant c reveals the presence of larger in-plane compressive strain as described in the following.

Figure 4(b) shows Raman spectra around the high-energy E_2_(high) mode and the whole Raman spectra of the samples were shown in Supplementary Figure S1. The E_2_(high) mode is sensitive to strain and has been extensively used to quantify the stress in GaN epilayers^36–38^. The E_2_(high) peaks for green LEDs with LT-GaN NL (19 nm) and with various thicknesses of sputtered AlN NLs (19, 26, 33, and 40 nm) on sapphire substrate were measured to be 569.07, 570.14, 569.47, 568.68, and 568.83 cm^−1^, respectively. The standard E_2_(high) peak of stress-free GaN bulk substrate is 566.65 cm^−1^. It is worth noting that the E_2_(high) peak of green LEDs on LT-GaN and sputtered AlN/sapphire templates redshifts with respect to the stress-free GaN bulk substrate, indicating the presence of in-plane compressive strain. Here, we derived the in-plane compressive stress of green LEDs by $\sigma (Raman)=\frac{{\rm{\Delta }}\omega }{4.2}GPa$, where Δ*ω* is the frequency shift of the GaN high-energy E_2_(high) mode peak relative to the stress-free GaN bulk substrate^38^. According to the Δ*ω*, the in-plane compressive stress of green LEDs with LT-GaN NL (19 nm) and with various thicknesses of sputtered AlN NL (19, 26, 33, and 40 nm) on sapphire substrate was calculated to be 576, 831, 671, 483 and 519 MPa, respectively.

Table 1 summarizes the results of XRD and Raman measurements. By comparing these measurement results, we found that the in-plane compressive stress of the green LED on sputtered AlN (19 nm)/sapphire template was larger than that of the green LED on LT-GaN (19 nm)/sapphire template. One explanation for this effect is that GaN films grown on LT-GaN NL (19 nm) are possibly more relaxed than that grown on sputtered AlN NL (19 nm), since the elastic constants of GaN NL are lower than those of sputtered AlN NL^39^. We also noted that the green LEDs on sputtered AlN/sapphire templates experience some levels of stress relaxations in different NL thicknesses. The compressive stress of green LEDs on sputtered AlN/sapphire templates decreased as the thickness of sputtered AlN NL increased from 19 nm to 33 nm but increased as the thickness of sputtered AlN NL increased from 33 nm to 40 nm. The significant stress relaxation occurs in the green LED on sputtered AlN (33 nm)/sapphire template having the minimum stress of 483 MPa, which exhibited a much smaller in-plane compressive stress than the green LED on LT-GaN/sapphire template. The difference in the measured in-plane compressive stress between XRD and Raman may be due to the uncertainty of the material constants.

**Table 1:** Material characterization results for green LEDs on LT-GaN and sputtered AlN/sapphire templates.

Sample	Lattice constant *c*	In-plane compressive strain (XRD)	In-plane compressive stress (XRD)	In-plane compressive stress (Raman)
Green LED with LT-GaN NL (19 nm)	5.189500 Å	0.171%	819 MPa	576 MPa
Green LED with sputtered AlN NL (19 nm)	5.190320 Å	0.202%	968 MPa	831 MPa
Green LED with sputtered AlN NL (26 nm)	5.189862 Å	0.185%	885 MPa	671 MPa
Green LED with sputtered AlN NL (33 nm)	5.189186 Å	0.159%	762MPa	483 MPa
Green LED with sputtered AlN NL (40 nm)	5.189316 Å	0.164%	786 MPa	519 MPa

**Figure 5:**
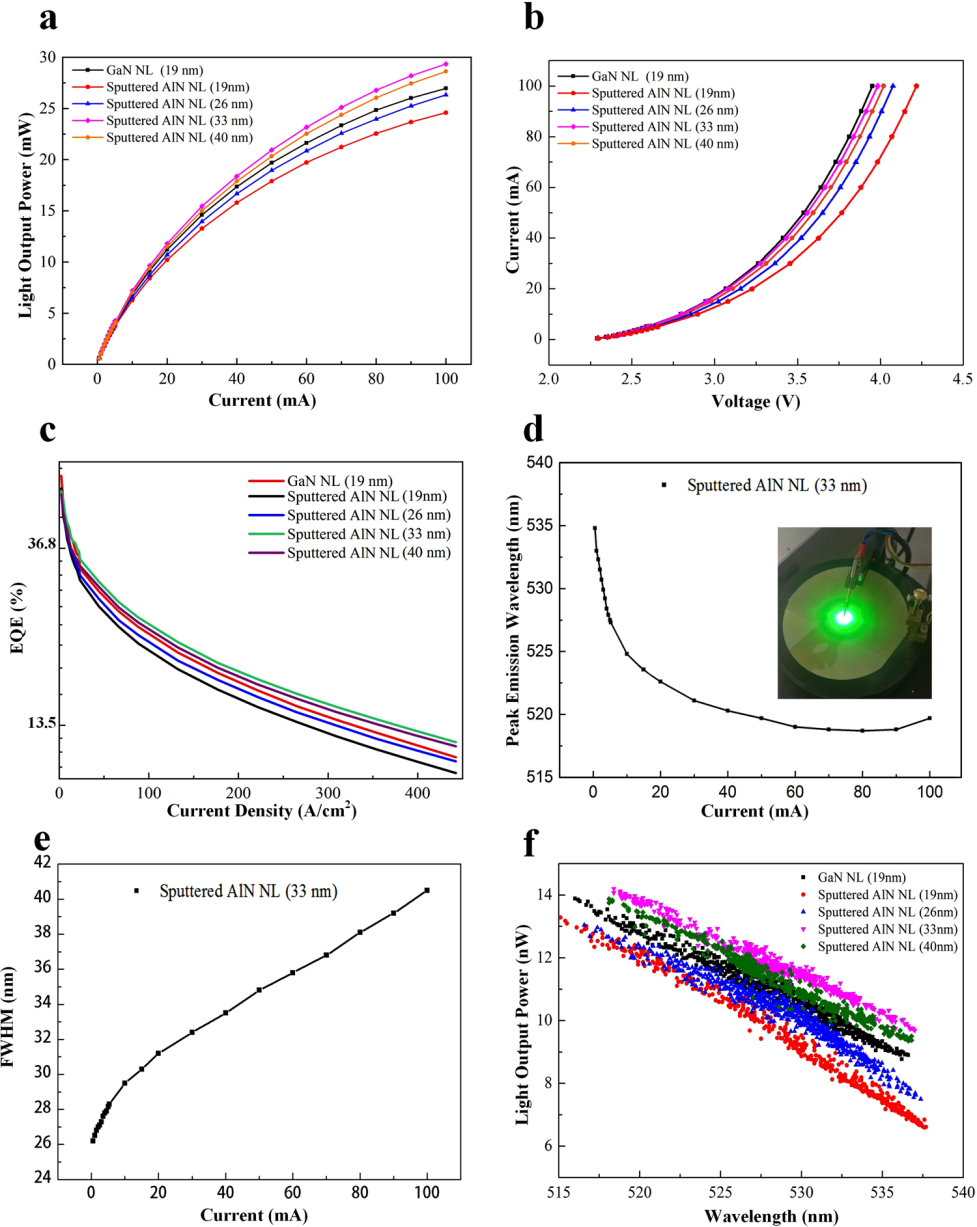
(**a**) Light output power versus current characteristics and (**b**) current versus voltage characteristics of green LEDs on LT-GaN and sputtered AlN/sapphire templates. (**c**) Dependence of EQE on the injection current density of green LEDs. (**d**) Peak emission wavelength and (**e**) FWHM versus injection current for the green LED on sputtered AlN (33 nm)/sapphire template. (**f**) Dependence of light output power on emission peak wavelength for green LEDs on LT-GaN and sputtered AlN/sapphire templates at 20 mA.

The light output power versus current and current versus voltage characteristics are presented in Fig. 5(a). At 20 mA, the light output powers of green LEDs on LT-GaN (19 nm) and sputtered AlN (19 nm)/sapphire templates are measured to be 11.2 and 10.2 mW, respectively, accompanied by forward voltages of 3.068 and 3.226 V. Although the TDD of green LEDs on sputtered AlN (19 nm)/sapphire template is lower than that of green LEDs on LT-GaN (19 nm)/sapphire template, the light output power of green LEDs on LT-GaN (19 nm)/sapphire template is larger than that of green LED on sputtered AlN (19 nm)/sapphire template. This result might be associated with the larger in-plane compressive strain in the green LED on sputtered AlN (19 nm)/sapphire template in comparison with the green LED on LT-GaN (19 nm)/sapphire template, as evidenced by XRD and Raman measurements in Fig. 4. At 20 mA, the light output powers of green LEDs with various thicknesses of sputtered AlN NL (19, 26, 33, and 40 nm) on sapphire substrates are 10.2, 10.7, 11.8, and 11.5 mW, accompanied by the forward voltages of 3.23, 3.16, 3.08, and 3.11 V. Notably, the light output power of green LEDs on sputtered AlN (33 nm)/sapphire template is the highest among these samples. Moreover, the light output power of green LEDs on sputtered AlN (33 nm)/sapphire template is 5.3% higher than that of green LEDs on LT-GaN (19 nm)/sapphire template at 20 mA, which is attributed to the reduced in-plane compressive stress and also to the improved crystal quality. The experimental results indicates that the strain-induced QCSE plays a dominant role in determining emission efficiency of green LEDs, and that the stress manipulation realized by changing the thickness of sputtered AlN NL is important to suppress the detrimental effect of the QCSE.

The dependence of EQE on the current density of green LEDs is illustrated in Fig. 5(b). The EQEs of the green LEDs with LT-GaN NL (19 nm) and with various thicknesses of sputtered AlN NL (19 nm, 26 nm, 33 nm, 40 nm) on sapphire substrates are determined to be 34.3, 32.1, 33.2, 36.4, and 35.2%, respectively, at injection current density of 20 A/cm^2^. Internal quantum efficiency (IQE) was determined by the time integrated PL intensity based on the assuming that the nonradiative recombination channels are suppressed at low temperature. Details on the IQE calculation were presented in the supplementary information (see Supplementary Figures S2–S6). IQEs of green LEDs with LT-GaN NL (19 nm) and with various thicknesses of sputtered AlN NL (19, 26, 33, and 40 nm) on sapphire substrates were estimated to be 41.9%, 38.3%, 41.0%, 44.97%, 42.7%, respectively. The trend of the EQE and IQE changes in green LEDs is consistent with that of the in-plane compressive stress measured for these green LEDs, indicating that the in-plane compressive stress in green LEDs plays a pivotal role in determining the quantum efficiency. The blue-shift of the peak emission wavelength and FWHM versus injection current of green LED on sputtered AlN (33 nm)/sapphire template is plotted in Fig. 5(c). The inset is the luminance image of the 4-inch green LED wafer at 10 mA, which renders a clear green emission. At 20 mA, the peak emission wavelength of green LED on sputtered AlN (33 nm)/sapphire template is 522 nm with a FWHM of 31 nm. As the injection current increases from 1 to 100 mA, the peak wavelength of the green LED on sputtered AlN (33 nm)/sapphire template shows a blue-shift as small as 13 nm. Meanwhile, the FWHM slightly rises from 26.6 to 39.9 nm by increasing current from 1 to 100 mA. Figure 5(d) shows light output powers of green LEDs as a function of emission peak wavelength at 20 mA. A typical ‘green gap’ phenomenon is clearly illustrated in Fig. 5(d), where the efficiency drops significantly as the wavelength increases. The green LED on sputtered AlN (33 nm)/sapphire template exhibited higher light output power than did the green LED on LT-GaN (19 nm)/sapphire template over the entire range of emission wavelengths, as shown in Fig. 5(d).

## Conclusion

We investigated the epitaxial growth of green LEDs on LT-GaN and sputtered AlN/sapphire templates. The green LED on sputtered AlN/sapphire template is superior to the green LED on LT-GaN/sapphire template in crystal quality. Moreover, we demonstrate that the in-plane compressive strain of green LEDs on sputtered AlN/sapphire template can be controlled by changing the thickness of sputtered AlN NL. For green LEDs on LT-GaN and sputtered AlN/sapphire templates, the green LED on sputtered AlN (33 nm)/sapphire template achieves the lowest in-plane compressive stress and the highest EQE (36.4%@20 A/cm^2^). Such a result can be attributed to the different effect of QCSE in the InGaN active layer induced by in-plane compressive stress. Hence, this work provides a straightforward method to achieve the purpose of manipulating in-plane compressive stress and improving crystal quality for practical use in realizing high-efficiency InGaN/GaN green LEDs on sputtered AlN/sapphire template.

## Methods

### Epitaxial growth and device fabrication

The 4-inch *c*-plane (0001) cone-shaped patterned sapphire substrates (PSS) was fabricated by combining a thermal-reflow photoresist technique and an inductively coupled plasma (ICP) etching. The bottom diameter, height, and interval spacing of the pattern is 2.7, 1.75, and 0.3 *μ*m, respectively. A NMC iTops A330 AlN sputter system was used to deposit AlN NLs with different thicknesses (19, 26, 33, and 40 nm) on the 4-inch *c*-plane cone-shaped PSS by feeding 120 sccm N_2_, 30 sccm He, and 1 sccm O_2_. An aluminum disk (99.999%) was used as sputtering target. For comparison, a 19 nm-thick LT-GaN NL was also grown on the 4-inch *c*-plane cone-shaped PSS at 530 °C by metal-organic chemical vapor deposition (MOCVD) after the substrate was thermally cleaned in a H_2_ gas flow at 1050 °C. Trimethyl- or triethylgallium, trimethylindium, and trimethylaluminum were used as precursor gases for Al, Ga, and In, respectively. Bis(cyclopentadienyl)magnesium and disilane were used as magnesium and silicon sources for p- and n-type doping, respectively. Ammonia (NH_3_) were used as group-V precursor.

Green LEDs on LT-GaN and sputtered AlN/sapphire templates were grown in a Veeco K465i reactor. The epitaxial structure of green LEDs on LT-GaN and sputtered AlN/sapphire templates was composed of a 2.0 *μ*m-thick undoped GaN layer at 1100 °C, a 56 nm-thick n-AlGaN layer ([Si] = 2 × 10^18^cm^−3^) at 980 °C, a 2.0 *μ*m-thick n^+^-GaN layer ([Si] = 1.5 × 10^19^cm^−3^) at 1100 °C, 6 periods of In_0.02_Ga_0.98_N (3 nm)/GaN (27 nm) superlattice at 900 °C, 9 pairs of In_0.25_Ga_0.75_N (3.5 nm)/GaN (14.5 nm) MQW, a 20-nm-thick p-type LT-GaN layer at 800 °C, a 36-nm-thick 6 periods of p-AlGaN/GaN SL ([Mg] = 2 × 10^19^cm^−3^) at 960 °C, a 200-nm-thick p-GaN layer ([Mg] = 7 × 10^19^cm^−3^) at 960 °C, and a 3-nm-thick p^+^-GaN layer ([Mg] = 3 × 10^20^cm^−3^) at 960 °C.

For fabrication of the green LED chips, a mesa depth of 1.5 *μ*m was defined to expose the n-GaN layer using ICP etching based on BCl_3_/Cl_2_ mixture gas. A 115-nm-thick ITO layer was deposited as a current spreading layer on the p-GaN layer. Thermal annealing was performed at 550 °C for 30 min in ambient N_2_ to obtain low-resistant ohmic contact between ITO and p-GaN. Cr/Pt/Au multilayers were evaporated on the ITO and the exposed n-GaN layer as p- and n-type electrode. The size of the green LED chips on the wafer was 127 × 178 *μ*m^2^.

### Characterization and measurement

Cross-sectional TEM samples were prepared by focus ion beam milling using Ga ions at 30 kV, and then micrographs were taken with an FEI Talos F200X system at 200 kV (FEI, Hillsboro, OR, USA). For the time-integrated PL measurements, we used the 405 nm line of a 5 mW semiconductor laser as an excitation source. The detection part of the PL system consisted of a charge-coupled device detector (Princeton Instruments PIX IS256, Trenton, NJ, USA) connected to a spectrometer (Princeton Instruments SP2500i, Trenton, NJ, USA). PL spectra were measured using a reflex confocal microscope to focus the laser beam on the sample to a spot size of 500 *μ*m in diameter. The PL signal was dispersed in a 50 cm monochromator with an 1800 grooves/mm grating and detected by charge coupled device detector. High-resolution XRD was performed with Cu K*α*1 radiation of wavelength 1.54056 Å using a PAnalytical X’Pert Pro MRD diffractometer with a mirror, a four-bounce asymmetric Ge (220) monochromator and a triple-bounce analyser. Micro-Raman measurements were conducted by a Renishaw InVia spectrometer with the 633 nm frequency laser as the excitation source. The sub-bandgap laser illumination and low laser power guarantee that there is a negligible laser absorption and no interference with the device operating during the measurement. A 100× objective (N.A. = 0.85) microscope objective was used for focusing the laser and collecting the Raman scattered photons. Before each measurement, the wavelength and intensity were calibrated by silicon standard using the calibration system. All the Raman spectra were acquired with a diffraction grating of 1800 line/mm and slit width of 65 *μ*m in back scattering geometry. The light output power versus current and the current versus voltage characteristics of the green LEDs were measured by using a home-built probe station system^40^.

## Supplementary information

Effect of strain relaxation on performance of InGaN/GaN green LEDs grown on 4-inch sapphire substrate with sputtered AlN nucleation layer
