# Supplementary material for: Effect of strain relaxation on performance of InGaN/GaN green LEDs grown on 4-inch sapphire substrate with sputtered AlN nucleation layer
Source: Sci Rep. 2019 Mar 5;9:3447. doi: 10.1038/s41598-019-40120-9 (PMC6401382; doi:10.1038/s41598-019-40120-9)
Supplement: Supplementary file 1 — Effect of strain relaxation on performance of InGaN/GaN green LEDs grown on 4-inch sapphire substrate with sputtered AlN nucleation layer [file 41598_2019_40120_MOESM1_ESM.doc]

**Effect of strain relaxation on performance of InGaN/GaN green LEDs grown on 4-inch sapphire substrate with sputtered AlN nucleation layer**

Hongpo Hu^1^, Shengjun Zhou^1,2,*^, Hui Wan^1^, Xingtong Liu^1^, Ning Li^1^, Haohao Xu^1^

^1^Center for Photonic and Semiconductor, School of Power and Mechanical Engineering, Wuhan University, Wuhan 430072, China

^2^State Key Laboratory of Applied Optics, Changchun Institute of Optics, Fine Mechanics and Physics, Chinese Academy of Sciences, Changchun 130033, China

*corresponding.zhousj@whu.edu.cn

In the hexagonal structure, group theory predicts eight sets of phonon normal modes at the Γ point, 2A1 + 2E1 + 2B1 + 2E2. Among them, one set of A1 and E1 modes are acoustic, while the remaining six modes, A1 + E1 + 2B1 + 2E2, are optical. One A_1_, one E_1_ and two E_2_ are Raman active and only the A_1_(LO) and E_2_ modes can be observed in the backscattering configuration when the laser light is incident along the normal direction of the GaN-based LED [1]. The typical Raman spectra for green LEDs on LT-GaN and sputtered AlN/templates in the wavenumbers range of 100 to 1000 cm^-1^ were shown in Figure S1. As illustrated in figure S1, there are three peaks located at about 144, 568, and 734 cm^-1^ corresponding to the E_2_ (low), E_2_ (high) and A_1_(LO) mode respectively [2,3].

**
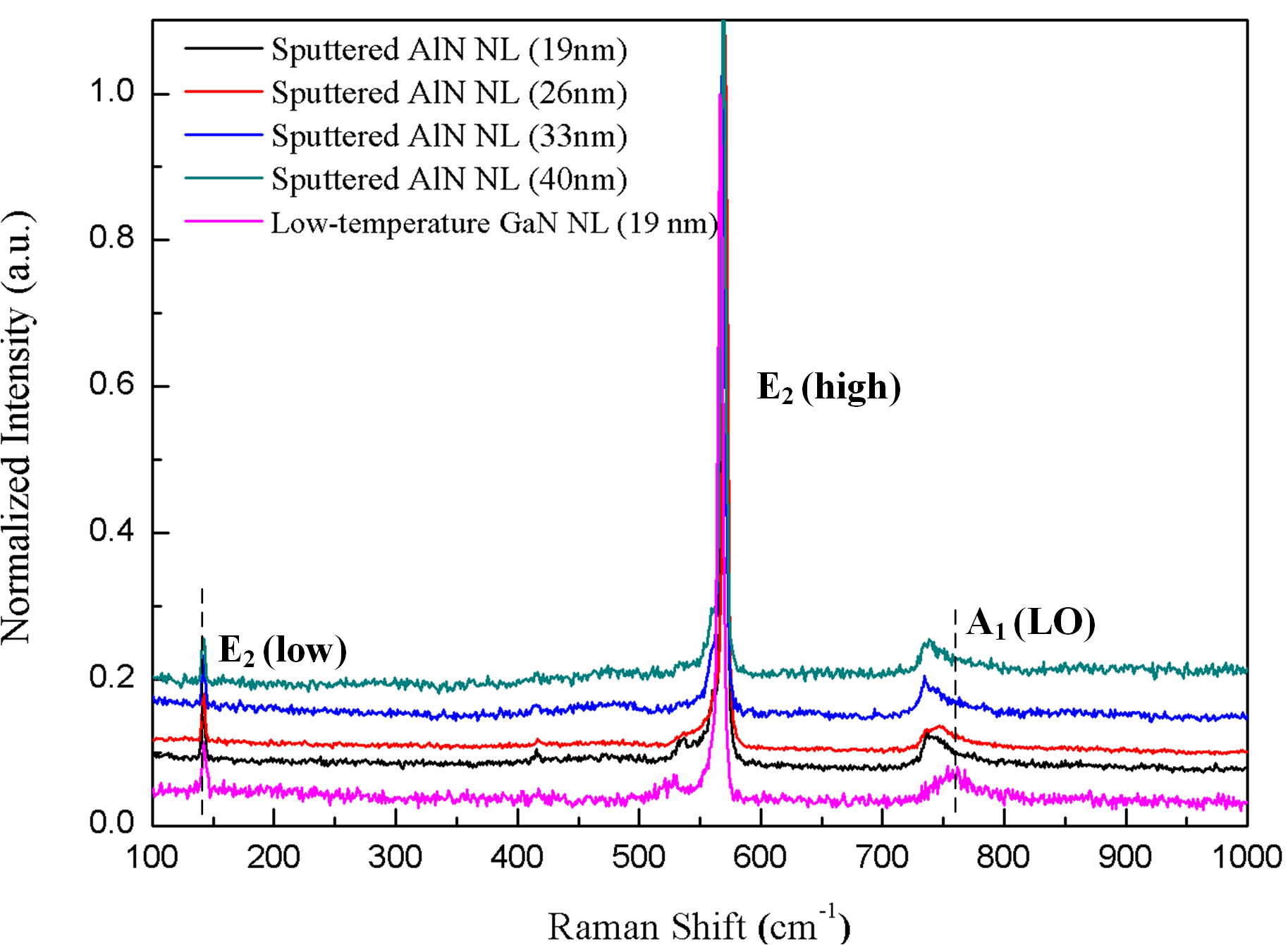
**

Fig. S1. Raman spectra for green LEDs on LT-GaN and sputtered AlN/sapphire templates.

Low-temperature (5 K) and room-temperature (300 K) photoluminescence (PL) measurements, as shown in Figs. S2-S6, were performed to estimate the internal quantum efficiency (IQE) of green LEDs on LT-GaN and sputtered AlN/sapphire templates using a 405 nm laser diode with an excitation power of about 5 mW. Assuming that the nonradiative recombination channels are suppressed at low temperature, the ratio of the spectrally integrated PL intensity at room temperature (300 K) to that at a low temperature (5 K) can be used to estimate the IQE of green LEDs. Here, the IQEs of green LEDs with LT-GaN NL (19 nm) and with various thicknesses of sputtered AlN NL (19, 26, 33, and 40 nm) LEDs were estimated to be 41.9%, 38.3%, 41.0%, 44.97%, 42.7%, respectively. The lower IQE for green LED on sputtered AlN (19 nm)/sapphire template than that for green LED on LT-GaN (19 nm)/sapphire template is attributed to the larger piezoelectric polarization field in QW.


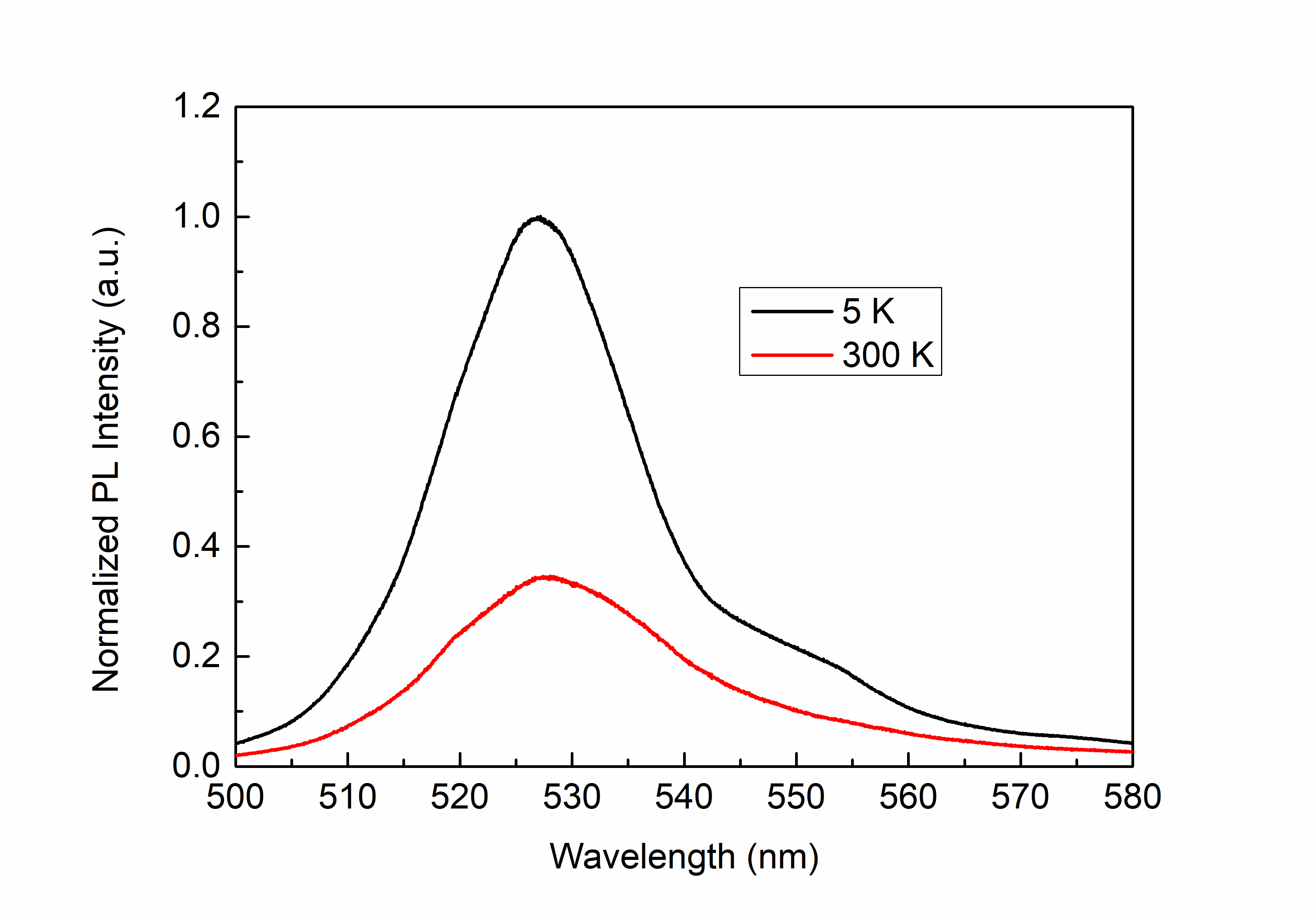


Fig. S2. PL spectra of the green LED on LT-GaN (19 nm)/sapphire template.


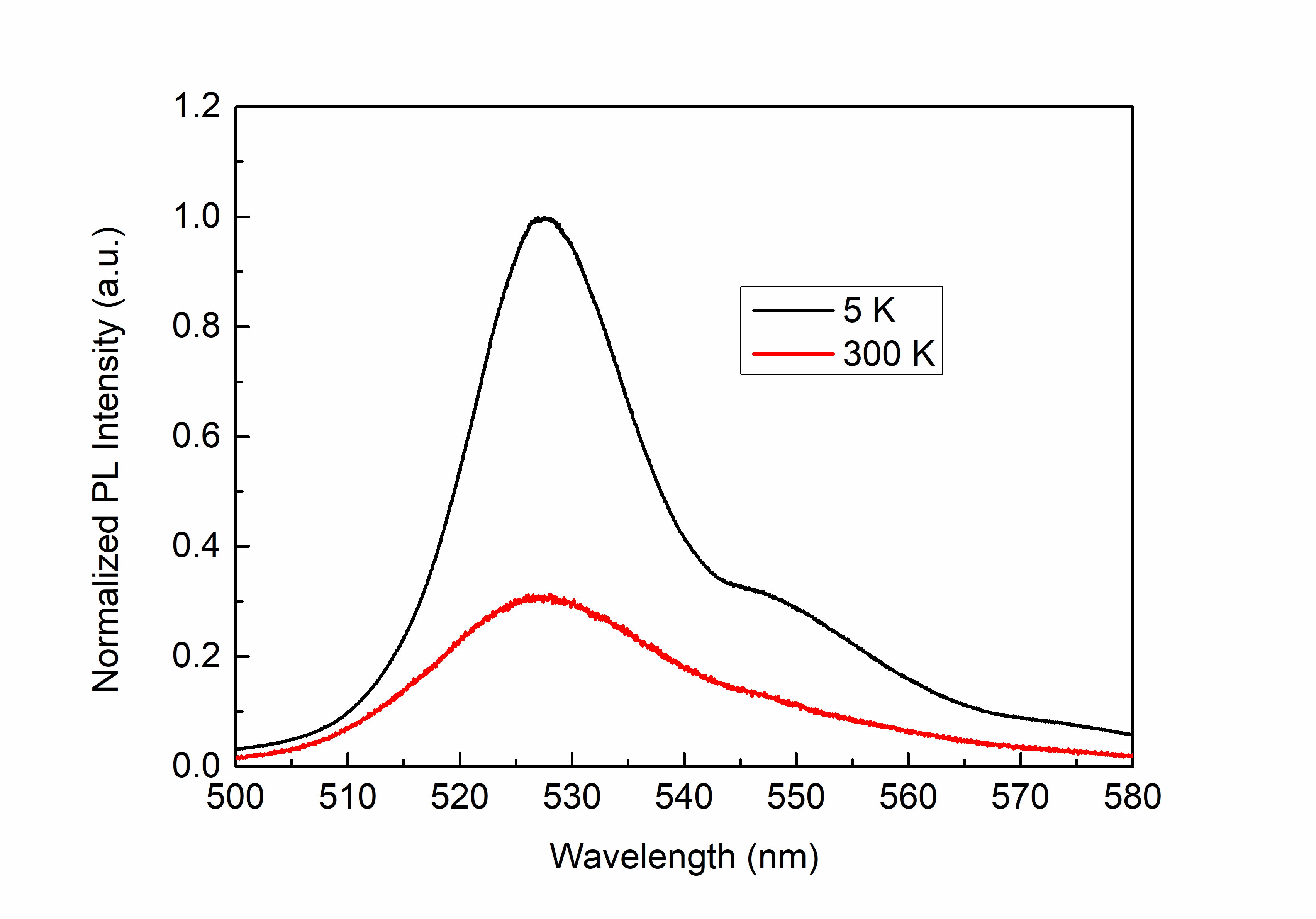


Fig. S3. PL spectra of the green LED on sputtered AlN (19 nm)/sapphire template.


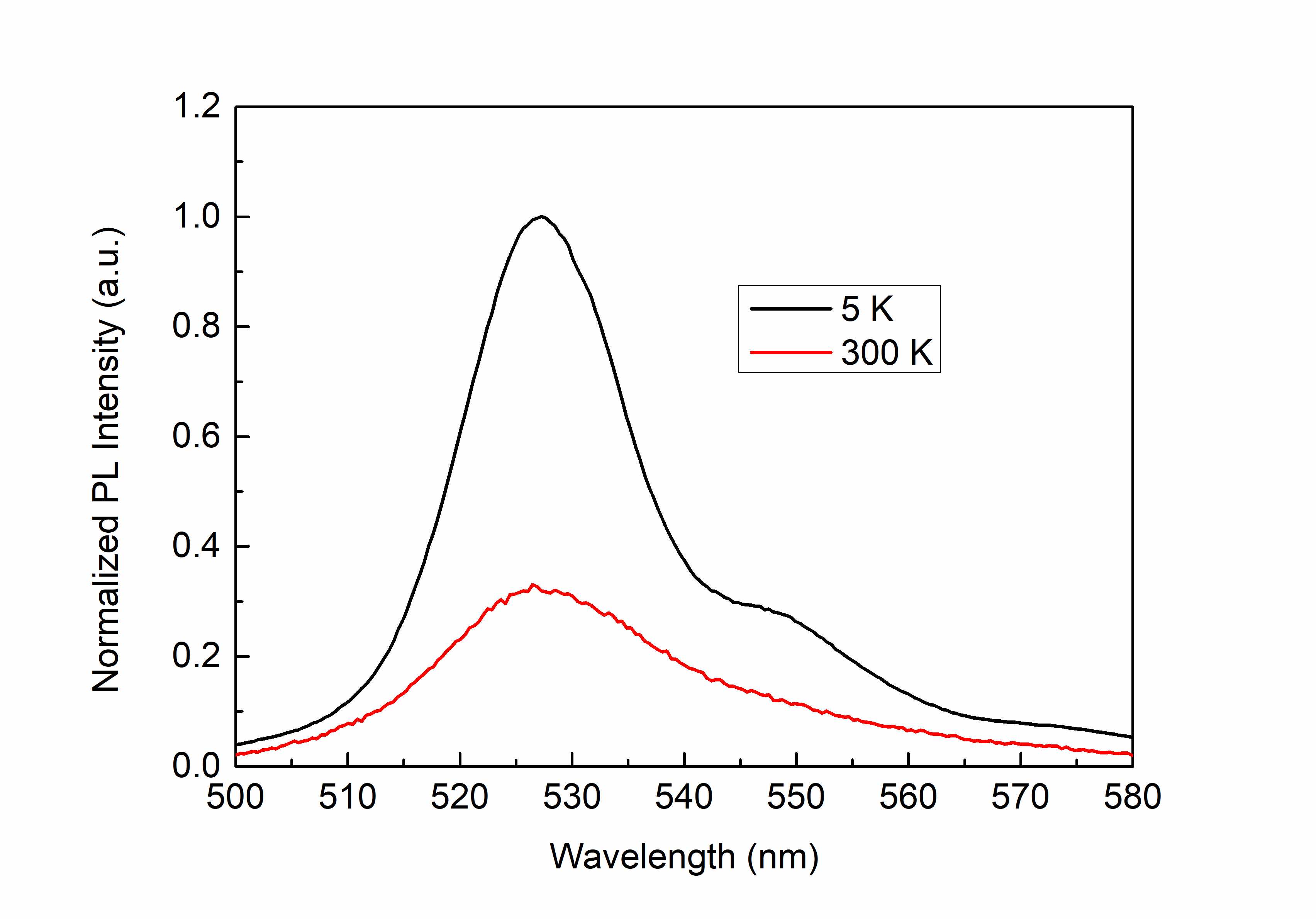


Fig. S4. PL spectra of the green LED on sputtered AlN (26 nm)/sapphire template.


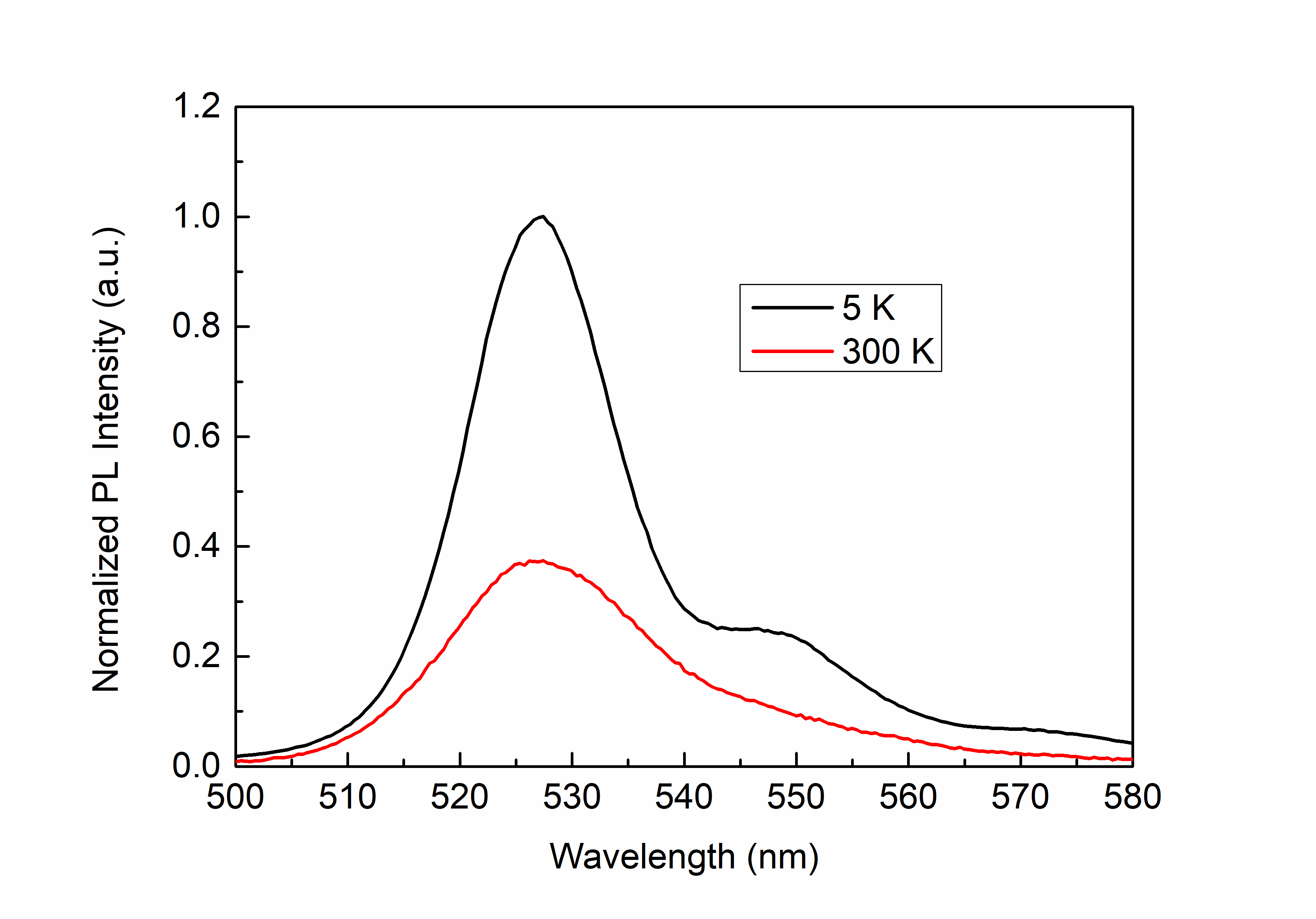


Fig. S5. PL spectra of the green LED on sputtered AlN (33 nm)/sapphire template.


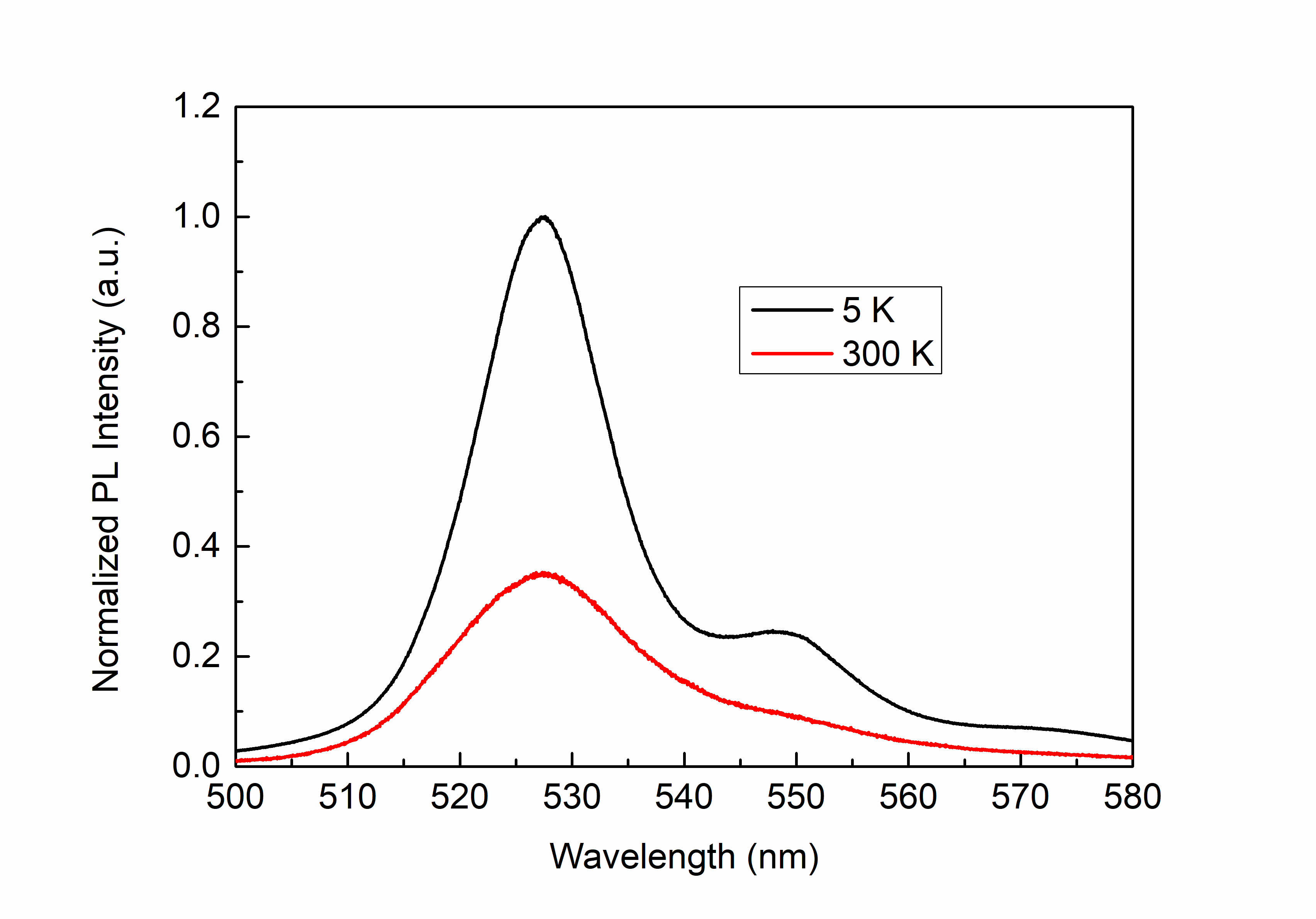


Fig. S6. PL spectra of the green LED on sputtered AlN (40 nm)/sapphire template.

**References**

1. Harima, H. Properties of GaN and related compounds studied by means of Raman scattering. *J. Phys.-Condes. Matter*, 2002, **14**(38): R967.

2. Matsui, R, Verma P, Ichimura T, et al. Nanoanalysis of crystalline properties of GaN thin film using tip-enhanced Raman spectroscopy. *Appl. Phys. Lett.*, 2007, **90**(6): 061906.

3. Feng, Z., Wang W., Chua S., et al. Raman scattering properties of GaN thin films grown on sapphire under visible and ultraviolet excitation. *J. Raman Spectrosc.*, 2001, **32**(10): 840-846.
